# Glucosinolate Diversity Analysis in Choy Sum (*Brassica rapa* subsp. *chinensis* var. *parachinensis*) Germplasms for Functional Food Breeding

**DOI:** 10.3390/foods12122400

**Published:** 2023-06-16

**Authors:** Seong-Hoon Kim, Parthiban Subramanian, Bum-Soo Hahn

**Affiliations:** 1 National Agrobiodiversity Center, National Institute of Agricultural Sciences, Rural Development Administration, Jeonju 5487, Republic of Korea; parthi@korea.kr (P.S.); bshahn@korea.kr (B.-S.H.); 2 Department of Physiology, Saveetha Institute of Medical and Technical Sciences (SIMATS), Saveetha Dental College & Hospitals, Saveetha University, Chennai 600077, India

**Keywords:** glucosinolate, germplasm, breeding, anticancer, genebank, diversity

## Abstract

The aim of this study was to analyze glucosinolates (GSLs) in germplasm that are currently conserved at the RDA-Genebank. The analysis focused on the glucosinolate diversity among the analyzed germplasms, with the goal of identifying those that would be most useful for future breeding efforts to produce nutritionally rich Choy sum plants. In total, 23 accessions of Choy sums that possessed ample background passport information were selected. On analyzing the glucosinolate content for 17 different glucosinolates, we observed aliphatic GSLs to be the most common (89.45%) and aromatic GSLs to be the least common (6.94%) of the total glucosinolates detected. Among the highly represented aliphatic GSLs, gluconapin and glucobrassicanapin were found to contribute the most (>20%), and sinalbin, glucoraphanin, glucoraphasatin, and glucoiberin were detected the least (less than 0.05%). We identified one of the accessions, IT228140, to synthesize high quantities of glucobrassicanapin and progoitrin, which have been reported to contain several therapeutic applications. These conserved germplasms are potential bioresources for breeders, and the availability of information, including therapeutically important glucosinolate content, can help produce plant varieties that can naturally impact public health.

## 1. Introduction

Choy sum (*Brassica rapa* subsp. *chinensis* var. *parachinensis*) is a leafy plant and is one of the representative horticultural crops widely consumed in Asian countries, including Malaysia, Cambodia, and China [1]. The leaves of the plant are used as food, and the plant grows swiftly to a height of 20–30 cm within a month, which makes it favorable to harvest edible leaves for cooking [2]. Choy sum is one of the representative *Brassica* crops widely used across Southeast Asian countries in their cuisine [3]. The flowers and peduncles are also consumed along with young leaves, and choy sum has been reported to be highly nutritious, containing 12 times more vitamin A, 2 times more vitamin C, 5 times more iron, and 1.5 times more calcium than Chinese cabbage (*Brassica rapa* subsp. *pekinensis)* [4,5,6,7].

In general, crucifers (*Brassica* genus) contain vitamins but are major sources of minerals and dietary fiber essential for the human body [4]. Moreover, plants from the family Cruciferae also synthesize metabolites called glucosinolates, which are anionic, hydrophilic secondary metabolites [8]. These Glucosinolates (GSLs) are synthesized by plants from the *Brassica* family (*Brassicaceae*) [9] and contain sulfur as well as nitrogen. Based on their R-side chain, they are divided into three categories: aliphatic, aromatic, and indolic GSLs. Each biosynthetic pathway of GSLs starts with an amino acid (AA), including methionine (met), phenylalanine (phe), and tryptophan (trp) as precursors [10]. After that, it goes through N-hydroxy-AA, aldoxime, and thiohydroximic acid to become desulfo GSL, and GSLs are finally synthesized through a series of processes [11]. Glucosinolates have been widely identified in Chinese cabbage (*Brassica rapa* subsp. *penkinensis*), cabbage (*Brassica oleracea*), broccoli (*Brassica oleracea* subsp. *italic*), etc., and are representative secondary metabolites of plants that also play a role in protecting plants from attacks by viruses or pests [12,13,14,15]. In terms of their value to humans, glucosinolates are potential compounds that have been well established for their anti-cancer as well as antioxidant functions [16,17]. 

The glucosinolate contents of *Brassica* vegetables have been of interest to researchers, and several reports exist on profiling glucosinolates from several plants [4,18,19]. However, reports on the glucosinolate compositions of conserved germplasm of *Brassica* are very scarce. Germplasm conserved in genebanks is a potential resource for breeders and scientists to generate plant varieties with desired characteristics, and information such as glucosinolate profiles would significantly help them choose appropriate germplasm. RDA-Genebank, as a representative national genebank in Korea, plays a crucial role in conserving a substantial collection of 11,000 accessions encompassing 83 subspecies of *Brassica*. The genetic resources held by RDA-Genebank exhibit remarkable abundance and diversity compared to the 3248 accessions conserved in ARS-GRIN [18]. In recent years, extensive profiling of GSLs has been conducted as a valuable dataset for conserved germplasm, which is readily accessible to researchers and breeders. Notably, GSL profiling specific to the Korean origins of Chinese cabbage involved the analysis of eight GSLs, with a focus on the inner, middle, and outer leaves [19]. Additionally, 10 GSLs were investigated for Chinese cabbage [20]. Furthermore, a selection of high-sinigrin germplasms in leaf mustard (*Brassica juncea*) underwent profiling for seven GSLs, and these germplasms were made available to breeders. It is worth mentioning that choy sum, classified under the *Brassica rapa* subspecies, remains unanalyzed for GSLs within international genebanks. To address this gap, a comprehensive profiling of 17 glucosinolates, including gluconapin, glucobrassicanapin, and progoitrin, which were previously recognized as major GSLs in *Brassica rapa*, was conducted with the utmost effort dedicated to enhancing the practical utility of the obtained results.

In choy sum, seedlings and microgreens have been reported to contain minerals, carotenoids, vitamins, and glucosinolates [4]. Screening of germplasms for their nutrient contents can provide potential candidates for the breeding of highly nutritional crop varieties. In the present study, we studied Choy sum germplasm available at the National Agrobiodiversity Center (RDA-Genebank) of the Rural Development Administration, Jeonju, Republic of Korea for their glucosinolate content.

## 2. Materials and Methods

### 2.1. GSLs Standards Used in This Experiment

All the reagents employed for both extraction and analysis in this study were analytical-grade products obtained from Sigma-Aldrich (St. Louis, MO, USA) and ThermoFisher Scientific Korea (Seoul, Republic of Korea). Among the 17 GSL standards, 6 GSLs including Progoitrin (PRO), Epiprogoitrin (EPI), Glucobrassicanapin (GBN), Glucoiberin (GIB), Glucoraphenin (GRE), and Sinalbin (SNB) were purchased from Phytolab (Martin Baue, KG, Germany) and the remaining 11 GSL were purchased from Phytoplan (Neuenheimer, Heidelberg, Germany). All standards had a purity of ≥98%.

### 2.2. Choy sum Genetic Materials and Cultivation Condition

Among the total 91 choy sum (*Brassica rapa* subsp. *chinensis* var. *parachinensis*) germplasms conserved at the National Agrobiodiversity Center (RDA-Genebank) Republic of Korea, the germplasms that lacked origin or status (landrace, cultivar, etc.) in their passport data were excluded, and 23 *Brassica rapa* subsp. *chinensis* var. *parachinensis* germplasms were selected as materials for our experiments [20]. The selected genetic materials, in order of the number of accessions, originated from Malaysia (5), Thailand (5), Taiwan (4), Vietnam (2), China (2), Mauritius (2), India (1), Laos (1), and Bangladesh (1). With regard to their biological status, they were segregated as landraces (17) and cultivars (6) (Table 1). The list of germplasms used in these experiments is detailed in Table S1. Since choy is an outcrossing crop, cross-fertilization was minimized by using a mesh material smaller than the size of the hole to minimize the scattering of pollen during the second generation (2019–2021) in the greenhouse from February to June. In addition, the multiplicated seeds were cultivated in the field from September to November, and heterogeneous germplasms were separated and continuously removed based on phenotype to maintain purity.

### 2.3. Sample Preparation: Pretreatment and Extraction

Leaves were harvested randomly from each plant in the accession. The harvested leaves were collected in polyvinyl bags and briefly stored at a temperature of −80 °C. Next, the leaves were lyophilized using an LP500 vacuum freeze-drier from Ilshinbiobase Co. in Dongducheon, Republic of Korea, for 2 days (48 h) and then ground into a fine powder. The harvested leaves were then moved back to −80 °C until profiling. The extraction of GSLs from the harvested leaves was performed using the method established earlier by Kim et al. in 2023. Specifically, 0.1 g of harvested leaves were mixed with 5 mL of 80% methanol and held at 25 °C for 30 min. Then, it was shaken continuously at 120 rpm for 30 min at 25 °C, followed by centrifugation of the mixture at 14,000 rpm for 10 min at 4 °C, and the supernatants were transferred to clean vials for further analysis [21].

### 2.4. Identification and Quantification of GSLs Using UPLC-MS/MS

The Acquity Ultra-Performance Liquid Chromatography, manufactured by Waters (Milford, CT, USA), coupled with the Xevo™ TQ-S system developed by MS Technologies (UK), was employed for the analysis of GSLs in accordance with the method described by Kim et al. (2023). In this experiment, a 5 µL sample was analyzed using an Acquity Ultra Performance Liquid Chromatography BEH C18 (1.7 µm, 2.1 × 100 mm) column (Waters Corp., UK). For elution, 0.1% trifluoroacetic acid in water was used as eluent A, and the eluent B mobile phase was 0.1% trifluoroacetic acid in methanol at a flow rate of 0.5 mL/min and 35 °C. The conditions for elution were set at 100% of A from 0.0 to 1.0 min, 100% of A from 1.0 to 7.0 min, 100–80% of A from 7.0 to 10 min, 80–0% of A from 10 to 11 min, 0–100% of A from 11 to 15 min, and 100% of A thereafter. Negative ion electrospray ionization and multiple reaction monitoring modes were used for the detection of the GSLs. The MS/MS parameters were set using capillary and cone voltages of 3 kV and 54 V, respectively, for ionization. The identification of detected GSLs was carried out by comparing their retention times and MS and MS/MS fragmentation spectra with those of commercially procured standards. Validation of the method’s precision and accuracy was performed by measuring linear, intraday, and intraday precision. To prepare the standards, 10 mg of individual GSLs were dissolved in methanol to obtain stock solutions (1 mg mL^−1^). Calibration curves were plotted using the corresponding standards to calculate GSL concentrations. The results were expressed as µmol GSLs kg^−1^ sample dry weight (DW). The limit of detection (LOD) and limit of quantification (LOQ) values were taken as three and ten times, respectively, the standard error of the intercept of the regression equation of the linear calibration curve divided by the slope. Fresh batches of test solutions were always prepared before sample analysis (Table 2).

### 2.5. Statistical Analysis

Pearson’s correlation is a widely used method for determining the relationship between two variables. Analyzed with XLSTAT software v2019 (Addinsoft, Paris, France), Depending on whether the relationship between the two variables was linear or non-linear, the strength and direction of the linear relationship between the two variables could be confirmed numerically. The Bartlett sphericity test confirmed the individual GSLs to be dependent on the others [22]. In addition, the results of our experiment with the Kaiser–Meyer–Olkin (KMO) test were used to confirm whether individual GSLs were suitable for PCA [21]. In this study, diversity was analyzed using 17 GSL profile values of 23 accessions of choy sum (*Brassica rapa* subsp. *chinensis* var. *parachinensis*) according to the method of Kim et al. [23,24].

## 3. Results and Discussion

### 3.1. Quantification of GSLs and Selection of Candidate Germplasm for Breeding Materials

**Figure 1:**
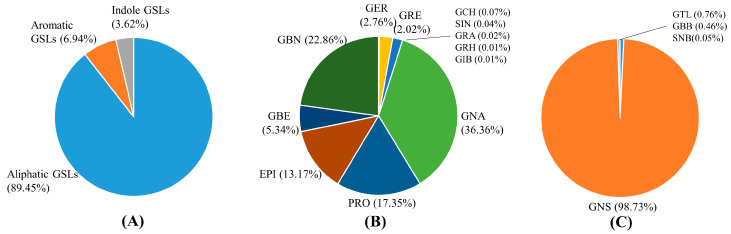
Proportion of the analyzed profiles in total glucosinolates; (**A**) 3 major pathways; (**B**) 12 aliphatic GSLs; (**C**) 4 aromatic GSLs in 23 choy sum germplasms. Progoitrin (PRO), Sinigrin (SIN), Gluconapin (GNA), Glucoiberin (GIB), Epiprogoitrin (EPI), Glucocheirolin (CGR), Glucoraphanin (GRA), Glucoraphenin (GRE), Glucobrassicanapin (GBN), Glucobarbarin (GBB), Glucoerucin (GER), Glucotropaeolin (GTL), Sinalbin (SNB), Glucoberteroin (GBE), Glucobrassicin (GBC), Gluconasturtiin (GNS), and Glucoraphasatin (GRH).

In this study, 17 glucosinolates of choy sum (*Brassica rapa* subsp. *chinensis* var. *parachinensis*) held at the RDA-Genebank were profiled using UPLC-MS/MS (Table 3). Overall, aliphatic GSLs were found to be high in choy sum leaves and ranged from 8243.00 to 18,110.85 µmol·kg^−1^ DW (average of 8243.54 µmol·kg^−1^ DW), taking up a vast majority (89.45%) of the total GSLs. Aliphatic GSLs were also predominantly found in our previous studies on Chinese cabbage (*Brassica rapa* subsp. *parachinensis*) [21]. Among the aliphatic GSLs, the gluconapin content ranged from 117.38 to 13,111.41 µmol·kg^−1^ DW, and the average was 2997.62 µmol·kg^−1^ DW, with total aliphatic GSLs contributing to the largest class of detected GSLs from choy sum at 36.36%. Next, the glucobrassicanapin content ranged from 148.87 to 6830.64 µmol·kg^−1^ DW, with an average of 1884.15 µmol·kg^−1^ DW. Detected quantities of progoitrin and epiprogoitrin ranged from 120.20 to 3172.65 µmol·kg^−1^ DW and 75.56 to 2728.20 µmol·kg^−1^ DW, respectively. Additionally, glucoberteoin indicated an average of 440.22 µmol·kg^−1^ DW, but some individual samples showed more than 3000 µmol·kg^−1^ DW in the results. The aromatic GSLs were detected at a range of 84.98 to 2389.73 µmol·kg^−1^ DW, accounting for 6.94% of the total GSLs. In particular, gluconasturtiin ranged from 74.28 to 2379.24 µmol·kg^−1^ DW, with an average of 651.28 µmol·kg^−1^ DW, accounting for the majority of the aromatic GSL. Finally, glucobrassicin, an indolic GSL was detected at an average of 333.30 µmol·kg^−1^ DW, accounting for 3.62% of the total GSLs (Figure 1). Several studies have been made on the GSL content of plants, especially *Brassica* vegetables [12,18,25,26,27]. Earlier studies on the GSLs from various rapa vegetables indicated that choy sum possessed specifically high levels of GNA among the GSLs compared to other vegetables of *Brassica* [18,25]. However, the least detected GSL was GBC in the case of He et al., 2003, against SNB observed in the current study [18]. The GSLs of *Brassica* vegetables seem to distantly vary both in total GSL content as well as individual GSLs produced [25]. For example, turnips, collards, pot herb mustard, and Chinese kale have been observed to contain the highest amount of total GSLs compared to Pak choi, Wutacai, turnip greens, and choy sum [25]. Among the individual GSLs, aliphatic GSLs are commonly high across *Brassica* crops, and among them, GNA and GBN are high in crops such as choy sum and Chinese cabbage, whereas GNA and PRO have been reported as the highest aliphatic GSLs in the case of Pak choy [21,26].

Our results aimed to provide breeders with a selection of potential candidate genetic resources for use in breeding programs to produce nutritionally enhanced natural foods. The germplasm with the highest total glucosinolate content (20,023.79 µmol·kg^−1^ DW) was found to be IT228140, which was introduced in 2003 to the RDA-Genebank from Myanmar Cultivar and is being conserved at the World Vegetable Center (AVRDC) (Table S1). In this particular germplasm, glucobrassicanapin (GBN) was at 6830.64 µmol·kg^−1^ DW, which was significantly higher than the average of 1884.15 µmol·kg^−1^ DW in this experiment. GBN’s hydrolysis product is 4-pentenyl isothiocyanate, which has been reported to increase antibacterial activity against *Aeromonas hydrophila*, a Gram-negative pathogenic bacteria [28]. It has also been found to decrease the release of leukotriene B4 (LTB4) from RBL in rats [29]. In addition, the progoitrin (PRO) content of IT228140 was also high at 3109.34 µmol·kg^−1^ DW. The hydrolysis product of PRO is nitrile crambene (1-cyano-2-hydroxy-3-butene), which has been reported to be effective in arresting the cell cycle of a hepatic cancer cell line [30]. However, PRO is directly related to the bitter taste of broccoli. So, crucifers containing PRO higher than 3000 µmol·kg^−1^ DW are known to be avoided by even animals due to their strong bitter taste, which limits their breeding potential [31].

### 3.2. Correlation Analysis

**Figure 2:**
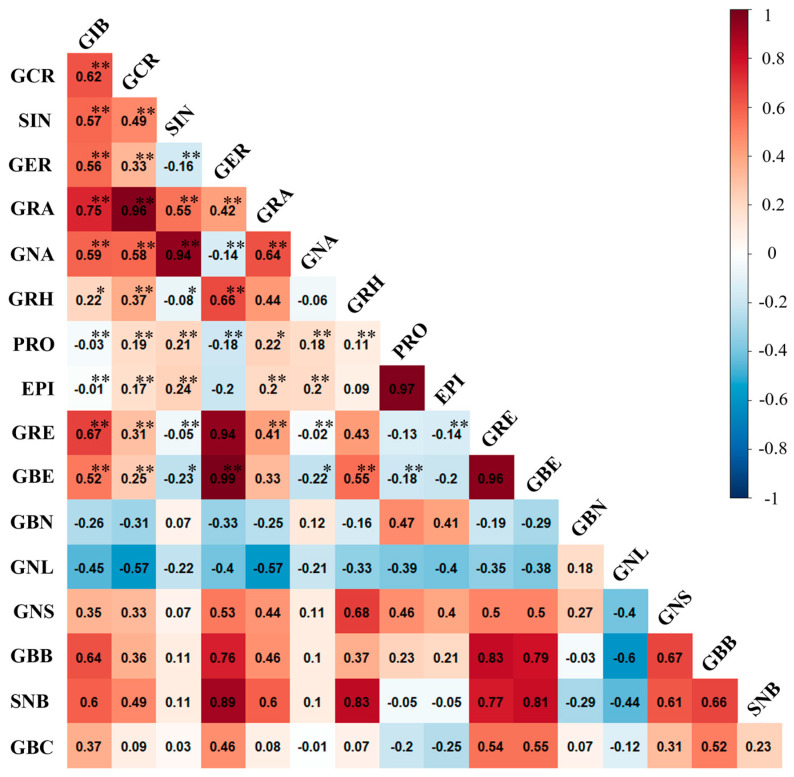
Pearson’s correlation analysis between individual glucosinolate compounds. *, ** Correlationship is significant at *p* ≤ 0.05 and *p* ≤ 0.01, respectively. Progoitrin (PRO), Sinigrin (SIN), Gluconapin (GNA), Glucoiberin (GIB), Epiprogoitrin (EPI), Glucocheirolin (CGR), Glucoraphanin (GRA), Glucoraphenin (GRE), Glucobrassicanapin (GBN), Glucobarbarin (GBB), Glucoerucin (GER), Glucotropaeolin (GTL), Sinalbin (SNB), Glucoberteroin (GBE), Glucobrassicin (GBC), Gluconasturtiin (GNS), and Glucoraphasatin (GRH).

**Figure 3:**
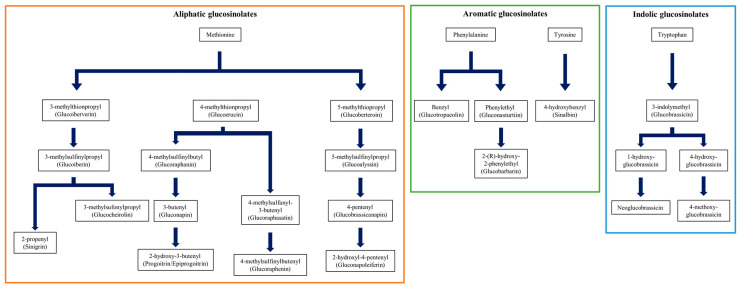
Three major biosynthesis pathways of glucosinolate in *Brassicaceae*. Flow charts explain the Aliphatic, Aromatic, and Indolic glucosinolate pathways [21].

Pearson correlation is a widely used method to determine the relationship between two variables. To investigate the relationship between individual glucosinolates in this experiment, we conducted a Pearson correlation analysis (Figure 2). Our results showed a highly positive correlation between gluconapin, a major GSL in Chinese cabbage (*Brassica rapa* spp.), and sinigrin, a major GSL in leaf mustard (*Brassica juncea*) (*r* = 0.939, *p* < 0.001). We also found sequential correlations between gluconapin, glucoiberin, and glucocheirolin (*r* = 0.636, *p* < 0.001; *r* = 0.589, *p* < 0.001; *r* = 0.579, *p* < 0.001, respectively). However, we could not observe any significant relationship between the remaining individual GSLs, including glucobrassicanapin, which is another major GSL in Chinese cabbage. In addition, we found that glucobrassicanapin (GBN), a known major GSL in cabbage, had significant correlations with progoitrin (PRO) and epiprogoitrin (EPI) (*r* = 0.473, *p* < 0.001; *r* = 0.409, *p* < 0.001). Furthermore, we confirmed significant correlations between progoitrin (PRO) and epiprogoitrin (EPI) with glucobrassicanapin (GBN), a major glucosinolate in cabbage (*r* = 0.473, *p* < 0.001; *r* = 0.409, *p* < 0.001). Notably, PRO and EPI are stereoisomers of each other and are both metabolite products of GNA (*r* = 0.973, *p* < 0.001). The aliphatic biosynthesis pathway, which is one of the three biosynthetic pathways of GSLs, produces three different compounds, namely glucoiberin (GIB), glucoerucin (GER), and glucoberteroin (GBE), from methionine depending on the position of the methyl group (Figure 3). From this pathway, we observed a strong positive correlation between glucobeteroin and glucoraphenin, which are known to be metabolite products of glucoerucin (GER) along with GER itself (*r* = 0.985, *p* < 0.001; *r* = 0.939, *p* < 0.001). Further, we observed a high correlation between sinabin, which is synthesized from tyrosine in the aromatic GSL biosynthetic pathway, and glucobarbarin, which is synthesized from phenylalanine (*r* = 0.893, *p* < 0.001; *r* = 0.761, *p* < 0.001). Additionally, a correlation was also found between GER and glucobrassicin, which are both synthesized in the indole GSL biosynthetic pathway (*r* = 0.462, *p* < 0.001).

### 3.3. Diversity Analysis and Clustering

Bartlett’s sphericity test and Kaiser–Meyer–Olkin (KMO) test are essential to determining whether individual GSLs are suitable for Principal Component Analysis (PCA) analysis. With Bartlett’s sphericity test at the *p*-value level of 0.05, the individual GSLs were correlated and sufficient for PCA. By performing the KMO test, 17 GSLs were confirmed to be 0.6 or higher, which was sufficient to perform PCA (Table S2). To analyze the data and determine the most relevant components with the largest variance, we utilized PCA, a widely used and popular clustering method. We first reduced the data dimensions to four principal components (PCs) using eigenvectors with values greater than or equal to 1. PC1 explained 41.50% of the total variance with an eigenvector of 2.65; PC2, PC3, and PC4 explained 23.13%, 14.11%, and 8.62% of the total variance, respectively. PC1 showed positive correlations with SNB (0.34), GER (0.33), GRE (0.32), and GBB (0.32) and a negative correlation with GNL (−0.24). PC2 showed strong positive correlations in the order of GNA (0.40), SIN (0.39), PRO (0.37), and EPI (0.37). PC3 was correlated with PRO and EPI, and PC4 showed correlations with GBC (0.55) and GRH (−0.38). We finally selected two PCs (PC1 and PC2) as the principal components for the PCA, as PC2 and PC3 can also explain the two variables of PRO and EPI equally well (Table 4). 

**Figure 4:**
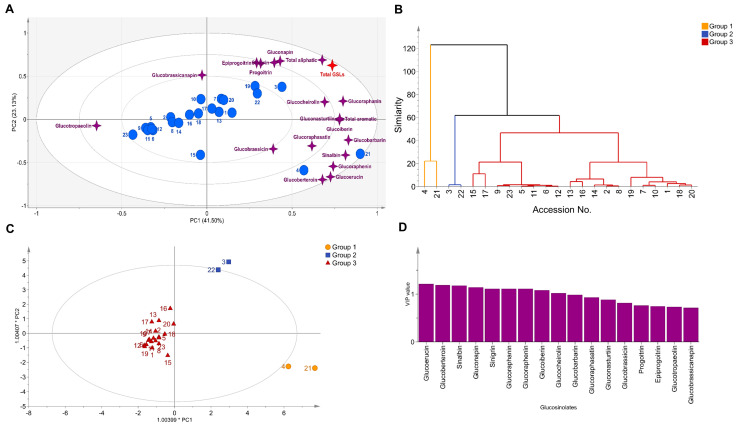
Diversity analysis among germplasms using individual GSL profile. (**A**) Principal Component Analysis (seventeen individual glucosinolates). (**B**) Cluster dendrogram, (**C**) Orthogonal Partial Least Squares Discriminant Analysis, (**D**) Variable Importance in Projection value.

Through PCA, using PC1 and PC2, we explain 64.63% of the total variance (Figure 4A). Clearly, the aliphatic GSLs had the greatest influence on total GSL content, especially with gluconapin, progoitrin, and epiprogoitrin. In contrast, germplasms with low total GSL were significantly influenced by the presence of glucotropaeolin, an aromatic GSL. The scatter plot of the germplasms indicated the possibility of the germplasms segregating into two clusters. PCA results have limitations in accurate clustering. Therefore, K-means clustering was applied to confirm optimal clustering. The optimal clustering was determined to be 3, and the results were visualized using a clustering dendrogram (Figure 4B). We employed Orthogonal Partial Least Squares Discriminant Analysis (OPLS-DA) to investigate the distribution reflecting the three clusters and identified the major variables that contributed to cluster differentiation using Variable Importance in Projection (VIP) values (Figure 4C). OPLS-DA showed clear clustering that could not be confirmed by PCA. Germplasms with high levels of glucoerucin, glucoraphenin, glucobeteroin, glucobarbarin, and sinalbin were included in group 1 (yellow dots). We also identified a new group 2 (blue squares), which showed a trend towards high total GSL along with high levels of gluconapin, glucoraphanin, glucoerucin, and sinigrin. The individual GSLs that contributed to the three clusters were largely influenced by VIP values, with glucoerucin, glucoberteroin, and sinalbin being the most influential, while glucobrassicanapin and glucotropaeolin were the least influential GSLs (Figure 4D).

### 3.4. Nutritional Value of Glucosinolates

Glucosinolates (GSLs) are secondary plant metabolites that, when acted upon by the enzyme myrosinase in the plant, produce isothiocyanates, which possess various physiological activities in both animals and humans (Table 5). In particular, it is widely known that GSLs induce the activity of phase 2 detoxification enzymes, which include glutathione-S-transferase, quinone reductase, and glucuronyl transferase, and exhibit various anti-carcinogenic functions [32,33,34,35]. The breakdown product of gluconapin, 1-cyano-3,4-epithiobutane, is known to prevent postprandial hypertriglyceridemia and reduce plasma triglyceride gain [36]. Our data reports high levels of GNA present in choy sum leaves (Figure 1, Table 3), which are a natural source of the therapeutic agent. Moreover, it has also been reported to increase the expression of NAD(P)H quinone oxidoreductase 1 (NQO1), glutathione S-transferase A3, and glutamate-cysteine ligase subunit (CETP) in Hep G2 cells, indicating its potential to induce diverse anti-carcinogenic effects [37]. Nitrile kramben, a degradation product of progoitrin, has been reported to increase the activity of quinone reductase in mouse Hepa 1c1c7 cells and mouse H4IIEC3 cells, as well as in human Hep Gsub2;/M phase, resulting in cell cycle arrest [38]. PRO is one of the top three GSLs detected in Choy sum leaves in the current study. Other studies have shown the therapeutic activities of GSL, such as protection against acute pancreatitis by inducing pancreatic acinar cell apoptosis in Swiss mice by activation of anti-inflammatory and mitochondrial pathways [38,39]. In our results, we found Choy sum to be enriched with GBN, which has been reported to have multiple potential benefits to human health. The compound, 4-Pentenyl isothiocyanate (4-PeITC), which is a degradation product of glucobrassicanapin, has been confirmed to have notable antibacterial activity against *Aeromonas hydrophila* (Gram-negative bacteria) and suppress the release of leukotriene B4 (LTB4) from RBL-2H3 (basophil leukemia cells) [28,29]. Indole-3-carbinol (I3C), a degradation product of glucobrassicin, inhibited glucocorticoid-induced osteoblast apoptosis by preventing the reactive oxygen species-mediated Nrf2 cycle in cells [40]. In the present study, we observed that the three GSL discussed above, GNA, GBN, and PRO were abundantly present in Choy sum naturally and could be recommended in diets for their antimicrobial and anticancer properties. Other potential GSLs have also been reported in other crops. It is well known that sulforaphane, a breakdown product of glucoraphanin in broccoli, has the potential to inhibit the development of prostate and lung cancer [41]. As a result, broccoli cultivars with high sulforaphane content have been selectively bred and commercialized. Glucobrassicin, which is hydrolyzed by myrosinase, produces not only sulforaphane but also indole-3-carbinol, which is known to suppress breast and ovarian cancer in humans [42]. Additionally, 2-phenylethyl isothiocyanate, a breakdown product of gluconasturtiin, is known to reduce cancer incidence by inhibiting the activity of phase 1 enzymes and promoting the induction of phase 2 enzymes [43]. Indole-3-carbinol is a derived product of glucobrassicin and sulphoraphane and remains one of the most potent anticancer compounds found in the GSLs in cruciferous vegetables [44]. Moreover, glucoiberin, progoitrin, and gluconasturtiin hydrolysis products have also been widely reported as inhibiting agents that protect human and animal cells against several carcinogens [45]. Overall, GSLs are of potential interest for human health due to their nutritional and therapeutic effects.

## 4. Conclusions

The study provides valuable information on the types and levels of GSLs produced by different germplasms of Choy sum. The data available through this research would be of potential interest for breeders to select candidate germplasm that exhibits desired desirable quantities of GSLs. From the results, we identified one accession, IT228140, to be rich in GBN and PRO, which have been extensively reported to contain antimicrobial, antitumor, and other therapeutic properties. Other germplasms also exhibit high amounts of GSLs and can be employed for breeding superior varieties of Choy sum with high GSL content. However, breeders should also consider the trade-off between glucosinolate content and palatability when selecting germplasm for breeding programs. The results of this study can help breeders select suitable germplasm with desirable glucosinolate content and taste for developing new cultivars with enhanced nutritional and health benefits.

## Figures and Tables

**Table 1:** Classification of origin and status of germplasms used in this experiment.

	Malaysia	Thailand	Taiwan	Vietnam	China	Mauritius	India	Laos	Bangladesh	Total
Cultivar	2	1	1	-	1	1	-	-	-	6
Landrace	3	4	3	2	1	1	1	1	1	17
Total	5	5	4	2	2	2	1	1	1	23

**Table 2:** UPLC spectroscopy information on seventeen glucosinolates studied in this experiment.

Name	Abbreviation	Class	RT (min)	MRM Transition	CID (ev)	Dwell Time (s)	Calibration Curve Parameters
Progoitrin	PRO	Aliphatic	5.94	387.77 > 194.85	25	0.029	Y = 8.2526X + 28.1501 (r^2^ = 0.961)
Sinigrin	SIN	Aliphatic	6.56	357.75 > 161.84	25	0.029	Y = 12.7878X − 11.1181 (r^2^ = 0.999)
Gluconapin	GNA	Aliphatic	7.78	371.74 > 258.74	25	0.029	Y = 8.36216X + 29.5397 (r^2^ = 0.994)
Glucoiberin	GIB	Aliphatic	7.98	421.62 > 357.73	25	0.029	Y = 33.6632X + 446.334 (r^2^ = 0.997)
Epiprogoitrin	EPI	Aliphatic	8.06	387.7 > 258.74	25	0.029	Y = 7.4939X − 6.76519 (r^2^ = 0.999)
Glucocheirolin	GCR	Aliphatic	8.38	437.71 > 258.74	25	0.029	Y = 20.7762X + 39.3608 (r^2^ = 0.986)
Glucoraphanin	GRA	Aliphatic	8.39	435.59 > 177.78	25	0.029	Y = 25.0808X + 60.584 (r^2^ = 0.983)
Glucoraphenin	GRE	Aliphatic	8.53	433.66 > 258.81	25	0.029	Y = 15.2565X + 3.62242 (r^2^ = 0.988)
Glucobrassicanapin	GBN	Aliphatic	8.60	385.71 > 258.87	25	0.029	Y = 7.2514X + 47.2841 (r^2^ = 0.992)
Glucobarbarin	GBB	Aromatic	8.64	437.71 > 274.75	25	0.029	Y = 9.29915X − 0.454779 (r^2^ = 0.999)
Glucoerucin	GER	Aliphatic	8.73	419.69 > 258.74	25	0.029	Y = 6.77393X + 73.6679 (r^2^ = 0.984)
Glucotropaeolin	GTL	Aromatic	8.88	407.72 > 258.87	25	0.029	Y = 18.2122X − 3.93949 (r^2^ = 0.999)
Sinalbin	SNB	Aromatic	9.10	423.62 > 258.74	25	0.029	Y = 49.7228X − 33.0636 (r^2^ = 0.999)
Glucoberteroin	GBE	Aliphatic	9.18	433.72 > 275.06	25	0.029	Y = 6.09397X + 63.1212 (r^2^ = 0.997)
Glucobrassicin	GBC	Indolyl	9.31	446.69 > 204.94	25	0.029	Y = 6.39827X + 2.6232 (r^2^ = 0.997)
Gluconasturtiin	GNS	Aromatic	9.34	421.69 > 274.87	25	0.029	Y = 4.36109X − 90.233 (r^2^ = 0.961)
Glucoraphasatin	GRH	Aromatic	9.62	417.63 > 258.81	25	0.029	Y = 15.5149X − 5.95281 (r^2^ = 0.997)

**Table 3:** Profile of individual glucosinolates in choy sum germplasms (μmol∙kg^−1^ DW).

Variable		Range	Mean	Std. Deviation
Aliphatic GSLs	Glucoiberin	0~1.48	0.39	0.46
	Sinigrin	0.16~17.69	3.69	4.21
	Glucocheirolin	0.08~19.91	5.48	6.07
	Glucoerucin	0.64~1983.01	227.83	562.36
	Glucoraphanin	2.29~569.16	166.77	179.00
	Gluconapin	117.38~13,111.41	2997.62	3406.77
	Progoitrin	120.20~3172.65	1430.06	899.82
	Epiprogoitrin	72.56~2728.20	1085.29	711.35
	Glucoraphasatin	0.03~9.89	0.70	2.02
	Glucoraphenin	0.11~9.18	1.35	2.10
	Glucoberteroin	6.02~3491.34	440.22	899.73
	Glucobrassicanapin	148.87~6830.64	1884.15	1457.35
	Total aliphatic	8243~18,110.85	8243.54	4557.95
Aromatic GSLs	Glucotropaeolin	1.83~9.58	4.87	2.08
	Gluconasturtiin	74.28~2379.24	631.28	575.41
	Glucobarbarin	0.97~8.04	2.94	1.71
	Sinalbin	0.04~2.96	0.34	0.69
	Total aromatic	84.98~2389.73	639.42	576.14
Indolic GSLs	Glucobrassicin	85.15~908.09	333.30	203.01
	Total GSLs	9216~20,023.79	9216.26	4905.73

**Table 4:** Four Principal components among 17 GSL in choy sum germplasms.

	Principal Component (Eigenvectors)			
	PC1	PC2	PC3	PC4
GIB	0.3	0.11	−0.27	0.19
GCR	0.25	0.25	−0.21	−0.22
SIN	0.09	0.39	−0.29	0.2
GER	0.33	−0.24	0.04	−0.03
GRA	0.28	0.25	−0.19	−0.17
GNA	0.1	0.4	−0.31	0.19
GRH	0.25	−0.06	0.18	−0.38
PRO	0.04	0.37	0.43	−0.03
EPI	0.03	0.37	0.41	−0.06
GRE	0.32	−0.2	0.01	0.18
GBE	0.31	−0.27	0.07	0.06
GBN	−0.09	0.17	0.36	0.44
GNL	−0.24	−0.17	−0.09	0.16
GNS	0.26	0.08	0.35	0.06
GBB	0.32	−0.03	0.16	0.23
SNB	0.34	−0.09	0.01	−0.19
GBC	0.17	−0.16	−0.02	0.55
Eigenvalue	2.65	1.89	1.55	1.21
Variability (%)	41.50	23.13	14.11	8.62
Cumulative (%)	41.28	64.63	78.74	87.36

**Table 5:** Functions of major glucosinolates and hydrolysis products analyzed in this experiment.

Chemical Compounds	Class	Hydrolysis Products	Functions
Gluconapin	Aliphatic	1-cyano-3,4-etithiobutane	**In Mice**
			Prevent postprandial hypertriglyceridemia and decrease plasma triglyceride gain [36]
			**In Human**
			Increase NAD(P)H quinone oxidoreductase 1 (NQO1), glutathione S -transferase A3 and the glutamate–cysteine ligase subunit (CETP) in Hep G2 Cell [46]
Glucobrassicanapin	Aliphatic	4-pentenyl isothiocyanate	**In Gram-negative bacteria**
			Increase antibacterial activity against Aeromonas hydrophila [28]
			**In rats** [29]
			Decrease release of leukotriene B4 (LTB4) from RBL
Progoitrin	Aliphatic	Nitrile Crambene (1-cyano-2-hydroxy-3-butene)	**In human Hep Gsub2 cell; mouse Hepa 1c1c7 cells and mouse H4IIEC3 cells** [38]
			Increase the activity of quinone reductase resulting in cell cycle arrest
			**In Swiss mice**
			In Swiss mice protect against acute pancreatitis by inducing pancreatic acinar cell apoptosis by activating anti-inflammatory and mitochondrial pathways [38,39] Decrease acute pancreatitis and activate anti-inflammatory pathway [38] Activate mitochondrial pathways [39]
Gluconasturtiin	Aromatic	2-phenylethyl isothiocyante	The anticancer activity of phenyl ethyl isothiocyanate, a hydrolyzed product obtained from gluconasturtiin, is excellent as it induces cytoprotective genes mediated by Nrf2 and AhR transcription factors, represses NF-κB, and inhibits both cytochrome P450 and histone deacetylase [30]
Glucobrassicin	Indolic	Indole-3-carbinol	**In human**
			Inhibit breast and ovarian cancer [47] Inhibit apoptosis of osteoporosis and ROS-mediated Nrf2 pathway [40]
			**In rat**
			Decrease portal hypertension, the severity of mesenteric angiogenesis, and portosystemic collaterals in cirrhosis [48]
			**In mice**
			Decrease *Citrobacter rodentium* growth causing acute intestinal inflammation and increase T cell activity [49]

## Data Availability

The data used to support the findings of this study can be made available by the corresponding author upon request.

## Supplementary Materials

The following supporting information can be downloaded at: https://www.mdpi.com/article/10.3390/foods12122400/s1, Table S1: Passport date of choy sum germplasms; Table S2: Kaiser–Meyer–Olkin measure of sampling adequacy.

## Abbreviations

GSL, glucosinolate; PRO, progoitrin; SIN, sinigrin; GNA, gluconapin; GIB, glucoiberin; EPI, epiprogoitrin; CGR, glucocheirolin; GRA, glucoraphanin; GRE, glucoraphanin; GBN, glucobrassicanapin; GBB, glucobarbarin; GER, glucoerucin; GTL, glucotropaeolin; SNB, sinalbin; GBE, glucoberteroin; GBC, glucobrassicin; GNS, gluconasturtiin; GRH, glucoraphasatin.
