# Supplementary material for: Glucosinolate Diversity Analysis in Choy Sum (Brassica rapa subsp. chinensis var. parachinensis) Germplasms for Functional Food Breeding
Source: Foods. 2023 Jun 16;12(12):2400. doi: 10.3390/foods12122400 (PMC10297880; doi:10.3390/foods12122400)
Supplement: Supplementary file 1 [file foods-12-02400-s001.zip › foods-2428586-supplementary.pdf]

Table S1. Passport date of choy sum germplasms.

Accessions No	RDA-No	Name	Statues	Origin
1	IT228139	CHOY SUM EX CHINA 1	Cultivar	MYS
2	IT228140	CHOY SUM EX CHINA 2	Cultivar	MYS
3	IT228155	40 DAYS RAPE	Cultivar	TWN
4	IT228157	ABD 5 BATON BLANC	Cultivar	MUS
5	IT228164	B01134	Landrace	IDN
6	IT259398	THA-SSJ-2002-6	Cultivar	THA
7	IT293077	6202	Landrace	THA
8	IT293078	7580	Landrace	CHN
9	IT297474	Kapi Shak	Landrace	BGD
10	IT301823	Xianggang cai xin	Cultivar	CHN
11	IT303512	40 Days Rape	Landrace	TWN
12	IT303513	Kra-Chon	Landrace	THA
13	IT303514	Cai Ngat	Landrace	VNM
14	IT303517	Phak Kat Moane	Landrace	LAO
15	IT303518	VI 033147	Landrace	MYS
16	IT303519	VI 033153	Landrace	MYS
17	IT306673	Abd 5 Bouquet	Landrace	MUS
18	IT306677	B 01019	Landrace	THA
19	IT306680	B 01024	Landrace	THA
20	IT306688	Cai Ngat	Landrace	VNM
21	IT306710	80 Days Rape	Landrace	TWN
22	IT306711	80 Days Rape	Landrace	TWN
23	IT306721	Green Petiole Var	Landrace	MYS

Table S.2. Kaiser-Meyer-Olkin measure of sampling adequacy

GIB	0.618
SIN	0.523
GCH	0.680
GER	0.692
GRE	0.650
GNA	0.546
PRO	0.514
EPI	0.540
GRH	0.751
GRA	0.617
GBE	0.586
GBN	0.551
GTL	0.534

GNS	0.610
GBB	0.604
SNB	0.629
GBC	0.546
KMO	0.601
